# SOX2 promotes hypoxia-induced breast cancer cell migration by inducing NEDD9 expression and subsequent activation of Rac1/HIF-1α signaling

**DOI:** 10.1186/s11658-019-0180-y

**Published:** 2019-08-22

**Authors:** Yueyuan Wang, Maria Bibi, Pengxiang Min, Wenjie Deng, Yujie Zhang, Jun Du

**Affiliations:** 1 Department of Physiology, Nanjing Medical University, 101 Longmian Avenue, Jiangning District, Nanjing, 211166 Jiangsu China; 2 Jiangsu Key Lab of Cancer Biomarkers, Prevention and Treatment, Collaborative Innovation Center for Personalized Cancer Medicine, Nanjing Medical University, 101 Longmian Avenue, Jiangning District, Nanjing, 211166 Jiangsu China

**Keywords:** Hypoxia, Migration, Breast cancer cells, SOX2, NEDD9

## Abstract

**Background:**

Hypoxia, a major condition associated with the tumor microenvironment, stimulates the migration of cancer cells. SOX2 is a powerful transcription factor that shows higher expression in several cancers, however, its role in hypoxia-induced breast cancer cell migration remains largely elusive.

**Methods:**

The human breast cancer cell lines MDA-MB-231 and MDA-MB-468 were cultured under hypoxic conditions. The cell migration rate was determined using the wound-healing and transwell assays. The protein levels of SOX2, NEDD9 and HIF-1α were evaluated via western blotting analysis. The NEDD9 mRNA levels were evaluated using qPCR. The activation of Rac1 was detected with the pulldown assay. The binding of SOX2 to the NEDD9 promoter was checked using the luciferase reporter assay. We also transfected breast cancer cells with specific siRNA for SOX2, NEDD9 or the Rac1 inactive mutant (T17 N) to investigate the role of SOX2, NEDD9 and Rac1 in the response to hypoxia.

**Results:**

Hypoxia markedly increased SOX2 protein levels in a time-dependent manner. SiRNA-mediated disruption of SOX2 inhibited cell migration under hypoxic conditions. Hypoxia also significantly augmented the NEDD9 mRNA and protein levels. Interestingly, SOX2 is a positive transcriptional regulator of NEDD9. Knockdown of SOX2 inhibited hypoxia-induced NEDD9 mRNA and protein expressions. Furthermore, hypoxia-induced upregulation of Rac1 activity and HIF-1α expression was attenuated by SOX2 or NEDD9 silencing, and Rac1-T17 N abolished HIF-1α expression as well as cell migration in cells subjected to hypoxia.

**Conclusions:**

Our results highlight the essential role of SOX2 in breast cancer cell motility. The upregulation of SOX2 under hypoxic conditions may facilitate NEDD9 transcription and expression, and subsequent activation of Rac1 and HIF-1α expression. This could accelerate breast cancer cell migration.

## Background

Breast cancer cell migration is controlled by various microenvironmental factors, such as cell–extracellular matrix interactions, secretory factors and the availability of oxygen, with hypoxia having considerable impact. Breast cancer cells incubated in hypoxic conditions are often associated with an aggressive metastatic phenotype showing increased resistance to clinical treatment [1–3]. A major aspect of the normal cell response to hypoxia is the upregulation of hypoxia inducible factor 1α (HIF-1α), which mediates significant transcription changes in several hundred genes [4]. Immunohistochemical studies have shown that increased HIF-1α protein levels are linked with increased risk of metastasis in breast cancer patients [5, 6], suggesting that HIF-1α may serve as a major accelerating factor for cancer cell migration under hypoxia.

SRY-related high-mobility groupbox 2 (SOX2) is a member of the SOX family of transcription factors. It regulates various cell functions, including differentiation, metabolism, inflammation, transformation and circadian clock function [7, 8]. It is well accepted that SOX2 can both directly bind to DNA targets to regulate the expression of related genes and form protein complexes that be used as transcriptional activators to maintain the undifferentiated state and self-renewal ability of embryonic stem cells [9]. SOX2 is widely expressed in skin, lung and mammary epithelial cells. Pathologically, SOX2 also shows higher expression in gastric, pancreatic, breast and other malignant tumors [10–13]. A recent study revealed that SOX2 is involved in promoting esophageal squamous carcinoma metastasis via modulation of slug expression leading to STAT3/HIF-1α signaling activation [14]. SOX2 was also shown to be relevant in the development of the stemness properties of breast cancer cells [15]. Targeting of SOX2 with miR-590-5p can inhibit breast cancer cell stemness and metastasis [16].

SOX2 is known to interact with HIF-1α. It enhances HIF-1α promoter activity to regulate glucose metabolism in gastric cancer [17]. Although a recent study showed that knockdown of HIF-1α decreased hypoxia-mediated SOX2 upregulation and prostate cancer cell invasion [18], the molecular link between SOX2 and HIF-1α in breast cancer cells under hypoxic conditions remains unclear. Our previous study demonstrated that hypoxia-induced HIF-1α expression in breast cancer cells involves a cascade of signaling events, including Rac1 activation [19]. Thus, it is worth exploring whether and how the Rac1/HIF-1α pathway is involved in SOX2-mediated breast cancer cell motility.

Neural precursor cell-expressed developmentally downregulated protein 9 (NEDD9) is a well-known scaffolding molecule for signaling proteins and it plays a significant role in cancer development [20, 21]. NEDD9 was found to be co-expressed with SOX2 in some tissues [22]. SOX2-deficient human glioma cells are ineffective at regulating NEDD9 expression and show impaired invasive proteolysis-dependent cell migration [23]. NEDD9 is also known to interact with HIF-1α. The hypoxia-mediated induction of NEDD9 expression in colorectal carcinoma cells significantly enhances HIF-1α transcriptional activity by modulating the interaction between HIF-1α and its transcriptional cofactor p300 [24].

Here, we find that upregulation of SOX2 facilitated hypoxia-induced breast cancer cell migration via regulation of NEDD9 transcription and expression. This then led to Rac1 activation and HIF-1α expression. Our results provide evidence that SOX2 is closely related with breast cancer cell migration in hypoxia and suggest it might be developed as a therapeutic target for breast carcinoma metastasis.

## Materials and methods

### Cell culture

Human breast cancer cell lines MDA-MB-231 and MDA-MB-468 were obtained from the Cell Biology Institute of the Chinese Academy of Sciences. The cells were cultured in HyClone Dulbecco’s modified Eagle’s medium (DMEM) high glucose (Thermo Fisher Scientific) supplemented with 10% (v/v) HyClone fetal bovine serum (FBS) in a humidified incubator at 37 °C with 5% CO_2_. Cells were grown on plastic dishes for protein extraction and wound-healing assays. pEGFP-N1 vector containing a dominant negative Rac1-T17 N insert was provided by Dr. Shoshana Ravid of the Hebrew University in Jerusalem, Israel. Cells were transfected with either pEGFP-N1 or pEGFP-N1 expressing Rac1-T17 N using Lipofectamine 2000 per the manufacturer’s instructions (Invitrogen).

Hypoxic conditions were maintained by exposing cells to a continuous flow of a humidified mixture of 1% O_2_, 5% CO_2_ and 94% N_2_ at 37 °C for the indicated time.

### Plasmids and siRNAs

China GenePharma synthesized the siRNAs specifically targeting SOX2 (1: 5′-CUGCAGUACAACUCCAUGATT-3′; 2: 5′-CCAUGGGUUCGGUGGUCAATT-3′; and 3: 5′-GCAGACUUCACAUGUCCCATT-3′) and NEDD9 (1: 5′-GAGGCGUUCAGUUUCUUGATT-3′; 2: 5′-CCAAGAACAAGAGGUAUAUTT-3′; and 3: 5′-GAUGGGAUCAACCGAUUGUTT-3′). Cells were transfected with siRNA duplexes using Lipofectamine 2000 (Invitrogen) according to the transfection method provided by the manufacturer. After transfection with siRNA for 48 h, the cells were cultured in hypoxic conditions for the indicated times.

### Cell wound-healing assay

For wound-healing assays, transfected cells were plated in six-well plates. When the cell confluence reached approximately 95–100%, a scratch was made using a 200-μl pipette tip. The wounded monolayer was washed with phosphate-buffered saline (PBS), then incubated in fresh medium with or without hypoxia. The wounded cellular monolayer was imaged 0 and 12 h after scratching using a Carl Zeiss Meditec microscope.

### Transwell assay

For migration assays, transfected cells were resuspended in 200 μl of serum-free DMEM and seeded at 4 × 10^4^ cells/well in the upper chamber of a Corning transwell plate with an 8.0-μm pore membrane. Cells were permitted to attach to the membrane for about 30 min. The lower chamber was filled with 600 μl DMEM with 10% FBS. After 12 h, the cells adhering to the chamber’s lower surface were fixed and cells remaining on the upper surface were removed. After staining in a dye solution containing 0.1% crystal violet for 5 min, the cells on the down surface of the membrane from five randomly selected high-power fields were counted under a Nikon TS100 microscope.

### Real-time PCR

Total RNA was extracted and purified using TRIzol reagent (Invitrogen) following manufacturer’s protocol. cDNA was synthesized using equal amounts of RNA (0.5 μg) from each sample. Quantitative PCR was performed using a GoTaq qPCR Master Mix assay (Promega) on the ABI StepOneTM Real-Time PCR System (Applied Biosystems). The primer sequences were: SOX2: 5′-GCCGAGTGGAAACTTTTGTCG-3′ (sense) and 5′-GGCAGCGTGTACTTATCCTTCT-3′ (antisense); NEDD9: 5′-GACCGTC ATAGAGCAGAACAC-3′ (sense) and 5′-TGCATGGGACCAATCAGAAGC-3′ (antisense); and β-actin: 5′-CATGTACGTTGCTATCCAGGC-3′ (sense) and 5′-CTCCTTAATGTCACGCACGAT-3′ (antisense). The gene expression level was calculated with Rt (2^-ΔΔCT^) values using StepOne Software v 2.1 (Applied Biosystems).

### Western blotting analysis

Whole-cell lysates were prepared in RIPA buffer (Beyotime). Sample protein extraction and concentration determination for whole cells were performed as previously described [25]. Briefly, equal amounts of protein were run on SDS polyacrylamide gels and transferred to nitrocellulose membrane. The resulting blots were blocked with 5% non-fat dry milk and probed with antibodies. The following antibodies were used: β-actin (Bioword), SOX2 (CST), NEDD9 (Santa Cruz), Rac1 (BD Biosciences) and HIF-1α (BD Biosciences). Appropriate secondary antibodies (Bioworld) were used at 1:20,000 dilutions, and the bands were visualized with ECL reagent (Millipore). Digital images of the positive bands were obtained and analyzed with Quantity One (Bio-Rad).

### Luciferase reporter assay

For the luciferase reporter assays, cells were seeded onto 24-well plates and transiently transfected with NEDD9 promoter reporter plasmid (Youbio) and siRNA targeting SOX2 using Lipofectamine 2000 for 48 h. Cells were collected and lysed for luciferase assays (Yeasen). Luciferase activity was measured using the Dual-Luciferase Reporter Assay System (Promega). Renilla luciferase was used for normalization. The transfection experiments were performed in triplicate for each plasmid construct.

### Pulldown assay

Rac1 activity was measured as previously described [26]. Briefly, 200 μg of total cellular protein was incubated with GST-PAK-CRIB fusion protein beads (donated by James E. Casanora of the University of Virginia) captured on MagneGST Glutathione Particles (Promega) for 4 h at 4 °C. The particles were then washed three times with washing buffer containing 4.2 mM Na_2_HPO_4_, 2 mM KH_2_PO_4_, 140 mM NaCl and 10 mM KCl (pH 7.2), resuspended in 2 x SDS sample buffer and subjected to western blotting analysis using a mouse anti-Rac1 antibody (BD Biosciences).

### Statistical analysis

All experiments were repeated at least three times and whole data are presented as means ± SD. Statistical analysis was carried out using the SPSS software. Student’s t test was used to analyze the differences between two groups. When comparisons between multiple groups were carried out, one-way ANOVA followed by SNK tests were employed. *p* < 0.05 represents statistical significance and *p <* 0.01 represents sufficiently statistical significance.

## Results

### SOX2 is essential for hypoxia-induced breast cancer cell migration

**Fig. 1:**
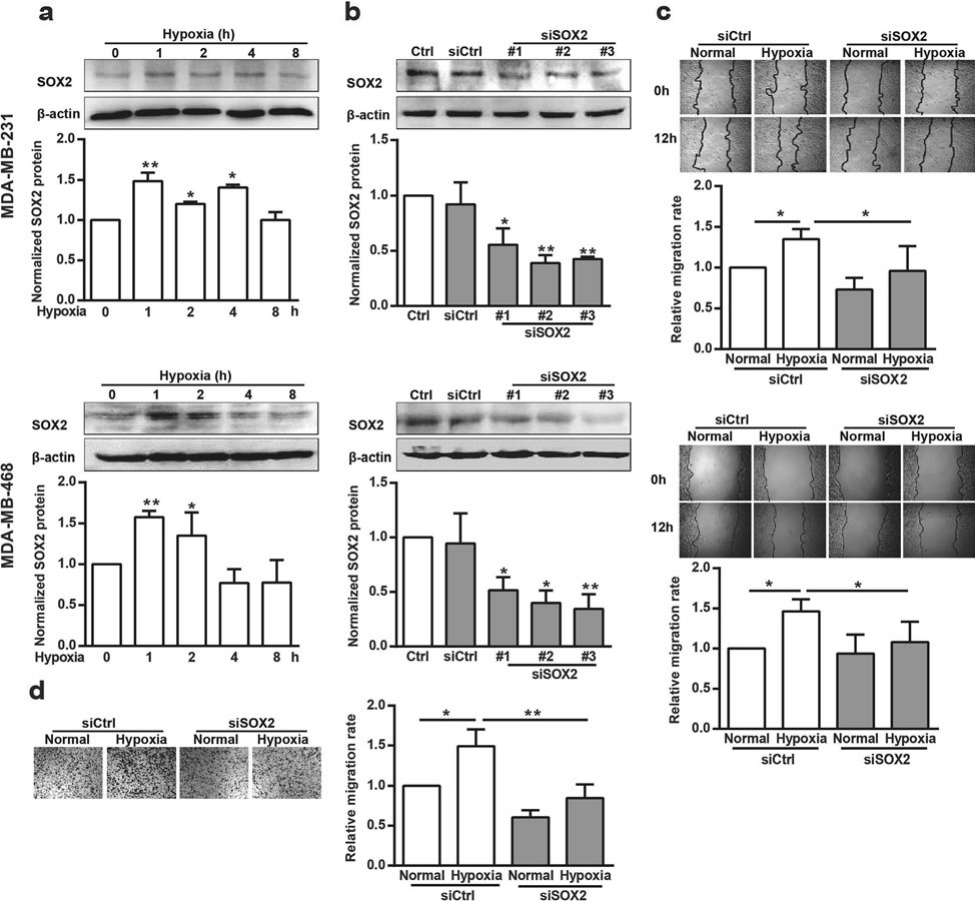
The effect of SOX2 on hypoxia-induced breast cancer cell migration. (**a**) MDA-MB-231 and MDA-MB-468 cells were incubated under hypoxia for the indicated times. Cellular lysates were assayed for SOX2 expression using western blotting. SOX2 was quantified and normalized against β-actin. *p < 0.05, **p < 0.01, referring to the difference between cells incubated with or without hypoxia. (**b**) Cells were transfected with siCtrl or siSOX2 for 48 h, then total protein extracts from cells transfected with siSOX2 were analyzed via western blotting for SOX2. SOX2 was quantified and normalized against β-actin. *p < 0.05, **p < 0.01, referring to the difference between cells treated with siCtrl or siSOX2. (**c**) The migratory capacity of cells transfected with siSOX2 under hypoxia for 12 h was evaluated using a wound-healing assay. (*n* = 10) *p < 0.05. (**d**) The migratory capacity of MDA-MB-231 cells transfected with siSOX2 under hypoxia for 12 h was evaluated using a transwell assay. *p < 0.05, **p < 0.01

To explore the role of SOX2 in mediating hypoxia-induced cell migration, we first tested whether hypoxia induced SOX2 expression in breast cancer cells. We found that the protein level of SOX2 increased in cells exposed to hypoxic conditions (Fig. 1a).

Then, we examined the effect of SOX2 on hypoxia-induced cell migration by knocking down SOX2 expression with appropriate siRNAs. Compared with siCtrl, siRNA 3 against SOX2 (3 siSOX2) most effectively reduced SOX2 protein expression in both MDA-MB-231 and MDA-MB-468 cells (Fig. 1b) and was selected for further experiments.

We also investigated cell migration using a wound-healing assay after transfecting these cells with siSOX2. The cell migration rate increased significantly in cells under hypoxic conditions compared with the rate for cells under normal conditions. However, in SOX2-silenced cells, this stimulatory effect of hypoxia on cell migration was greatly inhibited (Fig. 1c). MDA-MB-231 cell migration was also assessed using transwell migration assay, which showed similar results (Fig. 1d). These results indicate that the increased expression of SOX2 was essential for hypoxia-stimulated cell migration.

### NEDD9 stimulates cell migration under hypoxia

**Fig. 2:**
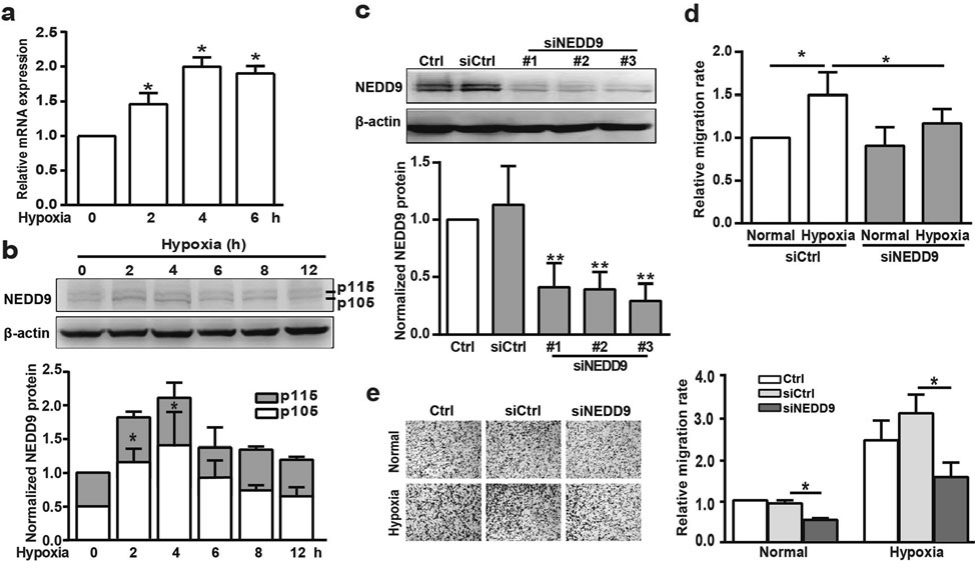
Effect of NEDD9 on hypoxia-induced breast cancer cell migration. (**a**&**b**) MDA-MB-231 cells were subjected to hypoxia for the indicated time and the NEDD9 mRNA or protein levels were determined using qPCR (**a**) or western blotting analysis (**b**). NEDD9 was quantified and normalized against β-actin. *p < 0.05 referring to the difference between cells incubated with or without hypoxia. (**c**) Cells were transfected with siCtrl or siNEDD9 for 48 h, then total protein extracts were analyzed via western blotting for NEDD9. NEDD9 was quantified and normalized against β-actin. **p < 0.01, referring to the difference between cells treated with siCtrl or siNEDD9. (**d**&**e**) The migratory capacity of those cells transfected with siNEDD9 under hypoxia was evaluated using a wound-healing assay (**d**) and transwell assay (**e**). The quantification of the cell migration rate was performed. *p < 0.05

To understand how SOX2 promotes breast cell migration, we set out to identify SOX2 target genes mediating its stimulatory activity. NEDD9, a noncatalytic scaffolding protein, contains docking sites for proteins involved in multiple signal transduction pathways. We measured NEDD9 mRNA and protein from MDA-MB-231 cells under control and hypoxic conditions. As shown in Fig. 2a and b, hypoxia treatment markedly increased the NEDD9 mRNA level, which was coincident with its protein expression. NEDD9 protein appeared as two phosphorylation-modified isoforms of 105 and 115 kDa. Incubation in hypoxia lowered the p115 isoform proportion compared to that for the control group, the proportion of the p105 isoform was higher in MDA-MB-231. Compared with siCtrl, siRNA 3 against NEDD9 (3 siNEDD9) effectively reduced NEDD9 protein expression (Fig. 2c). Consistently, knockdown of NEDD9 by 3 siNEDD9 completely blocked the effect of hypoxia on breast cancer cell migration, as assessed in the wound-healing and transwell migration assays (Fig. 2d and e).

### NEDD9 is a SOX2 target gene under hypoxia

**Fig. 3:**
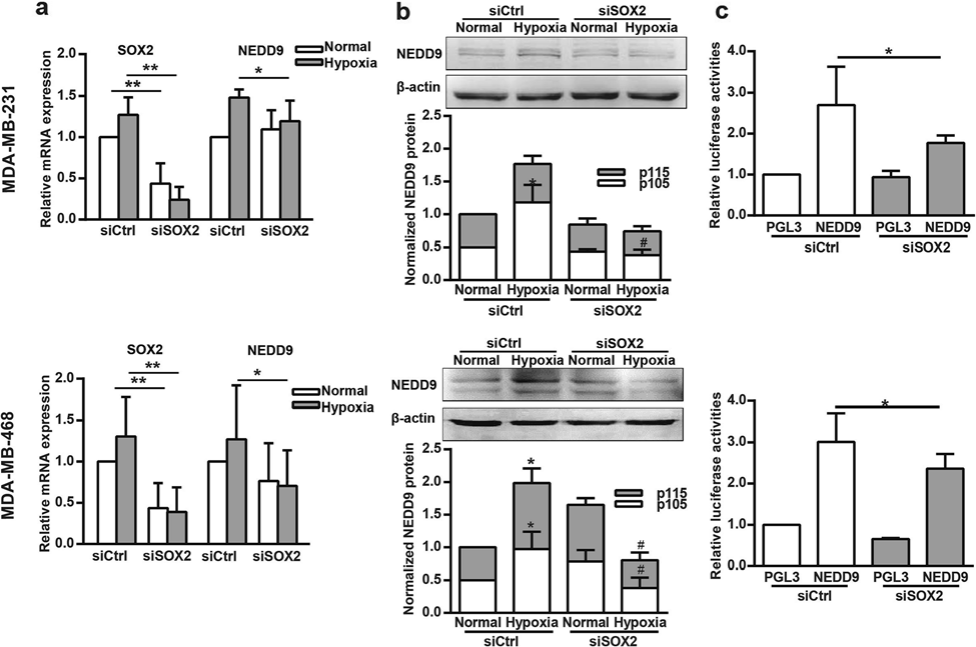
SOX2 is required for hypoxia-stimulated NEDD9 transcription and expression. (**a**&**b**) Cells transfected with siCtrl or siSOX2 were grown under hypoxia for 2 h, and NEDD9 mRNA (**a**) or protein levels (**b**) were examined via qPCR or western blotting analysis. In a, SOX2 and NEDD9 were quantified and normalized against β-actin. *p < 0.05, **p < 0.01. In b, NEDD9 was quantified and normalized against β-actin. *p < 0.05, referring to the difference between cells incubated with or without hypoxia. ^#^*p <* 0.05, referring to the difference between cells transfected with siCtrl or with siSOX2 under hypoxia. (**c**) Cells were co-transfected with the pGL3-Basic (control) or Luc-NEDD9 reporter and siCtrl or siSOX2, respectively. 48 h later, cell extracts were analyzed for luciferase activity. *p < 0.05

To further verify whether NEDD9 is regulated by SOX2 at the transcription level, we measured the NEDD9 mRNA and protein levels in SOX2-silenced MDA-MB-231 and MDA-MB-468 cells under hypoxia. Quantitative PCR and western blotting results showed that NEDD9 mRNA transcription and protein expression levels increased in hypoxia, but knockdown of SOX2 significantly reversed this upregulation (Fig. 3a and b).

We further amplified and cloned the NEDD9 promoter into the pGL3 luciferase vector. The luciferase reporter construct was co-transfected with siSOX2 into breast cancer cells. As shown in Fig. 3c, the activity of firefly/Renilla luciferase showed that knockdown of SOX2 drastically reduced the transcription driven by the NEDD9 promoter. These results indicate that expression of SOX2 is critical for the transcriptional activation and protein expression of NEDD9.

### Rac1 is required for SOX2- and NEDD9-mediated cell migration under hypoxia

**Fig. 4:**
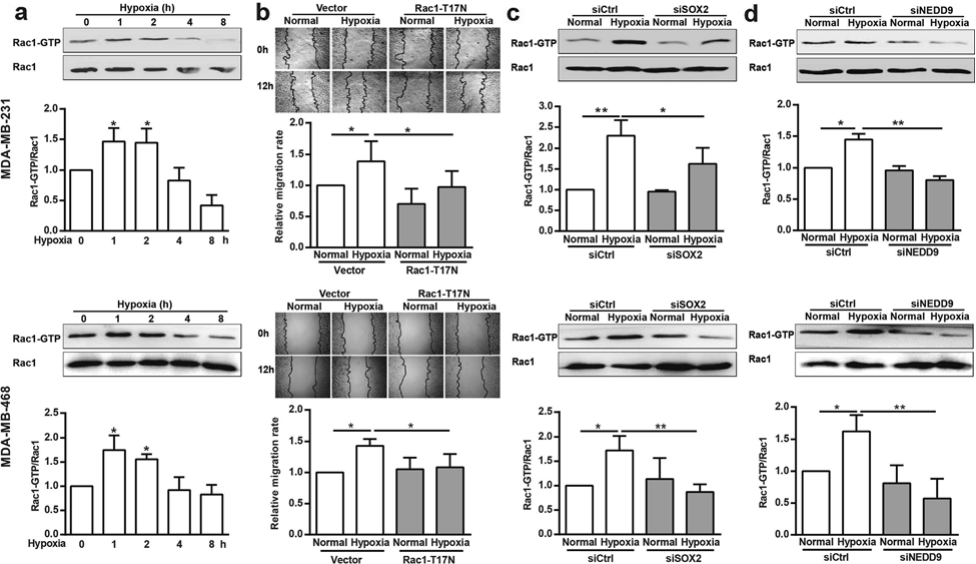
Rac1 is the downstream effector of SOX2- and NEDD9-induced breast cancer cell migration under hypoxia. (**a**) MDA-MB-231 and MDA-MB-468 cells were incubated with hypoxia for the indicated time and the induction of Rac1-GTP level was determined using pulldown analysis. The data were quantified and normalized against total Rac1. *p < 0.05, referring to the difference between cells incubated with or without hypoxia. (**b**) The migratory capacity of those cells transfected with empty vector or Rac1-T17 N plasmids under hypoxia was evaluated using a wound-healing assay. (*n* = 10). *p < 0.05. (**c**&**d**) After transfection with siSOX2 (**c**) or siNEDD9 (**d**), hypoxic cells were lysed and the Rac1-GTP level was determined via western blotting analysis. Rac1-GTP was quantified and normalized against Rac1. *p < 0.05, **p < 0.01

Previous reports have shown that hypoxia-induced breast cancer cell motility is Rac1 dependent and the Rac1 activity is driven by HIF-1α-mediated transcriptional induction of CXCR4 [27]. Here, we examined whether Rac1 is also involved in hypoxia-induced breast cancer cell migration. Hypoxia treatment in breast cancer cells led to the activation of Rac1 in a time-dependent manner (Fig. 4a), as determined with the pulldown assay. To further determine whether hypoxia stimulated breast cancer cell migration in a Rac1-dependent manner, we investigated cell migration using a wound-healing assay after transfecting these cells with Rac1-T17 N plasmids. Following incubation in hypoxic conditions, the cell migration rate increased significantly. However, in cells transfected with Rac1-T17 N, this stimulatory effect of hypoxia on cell migration was eliminated (Fig. 4b). Knockdown of SOX2 or NEDD9 also significantly reversed Rac1 activation induced by hypoxia (Fig. 4c and d). Collectively, these results show that Rac1 is required for SOX2- and NEDD9-mediated breast cancer cell migration under hypoxia.

### Hypoxia acts through SOX2, NEDD9 and Rac1 to promote HIF-1α expression

**Fig. 5:**
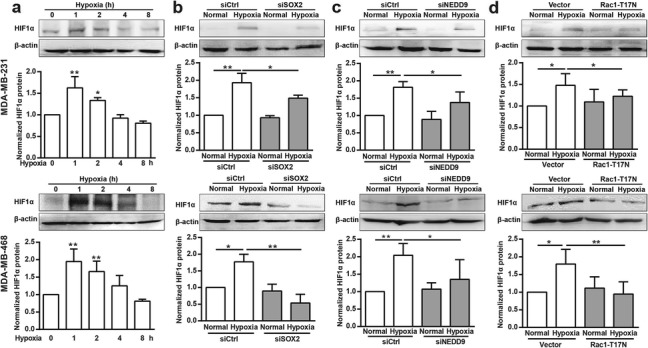
Rac1, SOX2 and NEDD9 are required for hypoxia-induced HIF-1α expression. (**a**) MDA-MB-231 and MDA-MB-468 cells were incubated under hypoxia for the indicated periods. Cellular lysates were assayed for HIF-1α expression via western blotting. HIF-1α was quantified and normalized against β-actin. *p < 0.05, **p < 0.01. (**b**-**d**) After transfection with siSOX2 (**b**), siNEDD9 (**c**) or Rac1-T17 N plasmids (**d**), hypoxic cells were lysed and HIF-1α expression was determined using western blotting analysis. HIF-1α was quantified and normalized against β-actin. *p < 0.05, **p < 0.01

Under hypoxia, multiple genes are upregulated by HIF-1α to initiate intracellular signaling pathways related with cell migration [28, 29]. As shown in Fig. 5a, HIF-1α was clearly augmented after hypoxia, and this was inhibited by knockdown of SOX2 (Fig. 5b) or NEDD9 (Fig. 5c), or by transfection with Rac1-T17 N (Fig. 5d).

**Fig. 6:**
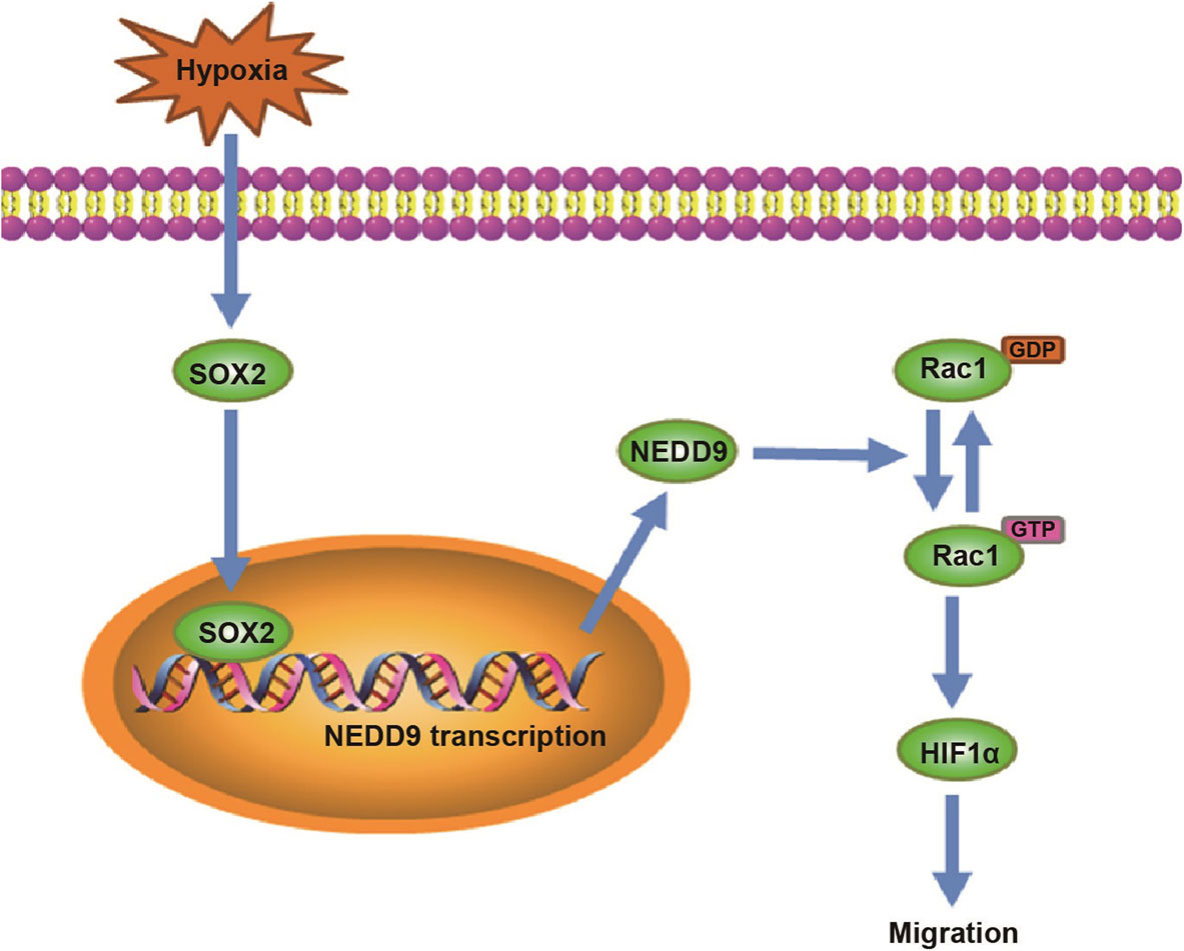
Illustration of the mechanism of SOX2 promotion of breast cancer cell migration. SOX2 potentiates breast cancer cell migration under hypoxia by supporting NEDD9 expression and leading to the activation of NEDD9 downstream effector HIF-1α signaling pathway. NEDD9 maintains HIF-1α protein stability under hypoxia, at least in part, in a Rac1-dependent manner

Our study demonstrated that hypoxia-induced HIF-1α expression involves a cascade of signaling events that involve SOX2 and NEDD9 and lead to subsequent Rac1 activation (Fig. 6).

## Discussion

Hypoxia is considered to play an independent role in tumor progression [30]. SOX2 is a stem marker found in cancer cells which could be upregulated under hypoxia [18, 31]. As in other studies, our primary observations are that hypoxia increases the SOX2 protein level in breast cancer cells and silencing of SOX2 suppresses the increased cell migration rate stimulated by hypoxia. These results indicated that increased SOX2 expression is critical for breast cancer cell migration in response to hypoxia.

In further investigations, we identified a novel link between SOX2 and NEDD9 in the regulation of breast cancer cell migration under hypoxia. NEDD9 is rarely mutated, but frequently shows elevated expression in cancer [20, 21]. A previous study demonstrated that NEDD9 is highly expressed in the hypoxic areas of human colorectal cancer specimens [24]. We noticed here that hypoxia induced NEDD9 expression in a time-dependent fashion in breast cancer cells. Normally, NEDD9 appears as two main phosphorylation-modified isoforms of 105 and 115 kDa [32]. The increase in the proportion of the 105 kDa isoform in MDA-MB-231 and 115 kDa isoform in MDA-MB-468 under hypoxia indicate a relative different increment of NEDD9 phosphorylation status in various types of breast cancer cells. We also observed that hypoxia not only enhances the NEDD9 protein level, but also increased its transcription activity. Knockdown of SOX2 significantly reversed the increased NEDD9 mRNA transcription and protein expression levels stimulated by hypoxia. Moreover, silencing of NEDD9 ameliorated hypoxia-stimulated breast cancer cell migration. These findings reveal that NEDD9 is a target gene for SOX2 and that it stimulates breast cancer cell migration under hypoxia.

Recent studies have shown that NEDD9 is involved in the control of cancer cell mesenchymal-mode movement in three-dimensional environments by affecting the Rac1 signaling cascade [33]. NEDD9 deficiency in cells could result in the acquisition of the amoeboid morphology, but it severely limits cell motility. Depletion of VAV2 was observed and that could impair the ability of NEDD9 to activate Rac1 [34]. In another study using a yeast two-hybrid screen, NEDD9 was also reported to mediate p75NTR-dependent Rac1 activation leading to cell spreading [35]. Therefore, it may be reasonable to speculate that the effect of NEDD9 on breast cancer cell migration is mediated by Rac1.

Rac1 belongs to a small GTPase family that exerts a specific regulatory role in cell motility. It participates in the control of the intracellular ROS production, which is implicated in HIF-1α signaling activation [36]. Previous results from our study and others showed that blocking Rac1 activation downregulated hypoxia-induced HIF-1α upregulation [19, 37]. It is therefore interesting to investigate whether Rac1 and HIF-1α work as downstream effectors of SOX2 and NEDD9 in hypoxic breast cancer cells. The results revealed that hypoxia triggers a slow increase in Rac1 activity and HIF-1α expression. Silencing SOX2 or NEDD9 blocks hypoxia-induced Rac1 activation, HIF-1α expression and cell migration. Our results also show that transfection of the inactive mutant form of Rac1-T17 N downregulated hypoxia-induced HIF-1α expression. These results indicate that SOX2 and NEDD9 play an important role in Rac1 activation and HIF-1α expression.

Elevated expression of SOX2 was reported to activate expression of the lncRNA PVT1, leading to breast cancer tumorigenesis [38]. The SOX2/miR-181a-5p, miR-30e-5p/TUSC3 axis is also identified as being closely linked with the proliferation and migration of breast cancer cells [39]. The upregulation of SOX2 following increased NEDD9 transcription under hypoxia leads us to conclude that hypoxia-induced HIF-1α expression and breast cancer cell migration at least in part, involves a cascade of novel signaling events, including SOX2 expression, activation of NEDD9 transcription and expression, and subsequent activation of Rac1.

These findings emphasize the pathophysiological importance of SOX2 as a potential therapeutic target for the treatment of breast cancer. It is noteworthy that both NEDD9 and SOX2 are recognized as HIF-1α downstream genes where they also participate in the control of cancer cell migration [18, 24]. Our study is the first to reveal that SOX2 and NEDD9 may function as novel upstream regulators of Rac1/HIF-1α in hypoxic breast cancer cells. We speculate that this positive feedback loop might contribute to adaptive and migratory responses of breast cancer cells encountering hypoxia.

## Conclusions

This study reveals SOX2 as a critical positive regulator of breast cancer cell migration under hypoxia. It could facilitate NEDD9 mRNA transcription and protein expression, and subsequent activation of Rac1/HIF-1α signaling and cell migration.

## Data Availability

Any information used and/or analyzed during this study is available from the corresponding author on reasonable request.

## Abbreviations

DMEM: Dulbecco’s modified Eagle’s medium
FBS: fetal bovine serum
HIF-1α: hypoxia inducible factor 1α
NEDD9: Neural precursor cell-expressed developmentally downregulated protein 9
PBS: phosphate-buffered saline
SOX2: SRY-related high-mobility groupbox 2
